# Oral Human Papillomavirus Infection in Men Who Have Sex with Men: A Systematic Review and Meta-Analysis

**DOI:** 10.1371/journal.pone.0157976

**Published:** 2016-07-06

**Authors:** Eleanor M. King, Soonita Oomeer, Richard Gilson, Andrew Copas, Simon Beddows, Kate Soldan, Mark Jit, W. John Edmunds, Pam Sonnenberg

**Affiliations:** 1 Research Department of Infection and Population Health, University College London, WC1E 6JB, London, United Kingdom; 2 The Mortimer Market Centre, Central and North West London NHS Foundation Trust, WC1E 6JB, London, United Kingdom; 3 Virus Reference Department, Public Health England, 61 Colindale Avenue, NW9 5EQ, London, United Kingdom; 4 Centre for Communicable Disease Surveillance and Control (CIDSC), Public Health England, 61 Colindale Avenue, NW9 5EQ, London, United Kingdom; 5 Modelling and Economics Unit, Public Health England, 61 Colindale Avenue, NW9 5EQ, London, United Kingdom; 6 Department of Infectious Disease Epidemiology, London School of Hygiene & Tropical Medicine, Keppel Street, WC1E 7HT, London, United Kingdom; Asociacion Civil Impacta Salud y Educacion, PERU

## Abstract

**Background:**

The epidemiology of oral human papillomavirus (HPV) infection in men who have sex with men (MSM) differs from anogenital HPV infection. The impact of HPV vaccination has, to date, largely focussed on anogenital outcomes. Vaccination of MSM in the UK has been recommended and, if implemented, baseline estimates of oral HPV prevalence will be useful.

**Methods:**

We searched Medline, Embase and psycINFO databases for studies reporting prevalence, incidence, and clearance of oral HPV infection in MSM. We performed a random-effects meta-analysis and meta-regression on prevalence estimates and summarised within-study risk factors for oral HPV DNA detection and incidence/clearance rates. We also performed a meta-analysis of the effect of MSM on oral HPV prevalence compared to heterosexual men.

**Results:**

26 publications were identified. The pooled prevalence of oral HPV16 from twelve estimates was 3.0% (95%CI 0.5–5.5) in HIV-negative and 4.7% (95%CI 2.1–7.3) in HIV-positive MSM. Median age of study participants explained 38% of heterogeneity (p<0.01) in HPV prevalence estimates (pooled = 17% and 29% in HIV-negative and HIV-positive, respectively; 22 estimates). Nine studies compared MSM to heterosexual men and found no difference in oral HPV prevalence (pooled OR 1.07 (95%CI 0.65–1.74)). The clearance rate was higher than incidence within studies. Type-specific concordance between oral and anogenital sites was rare.

**Conclusion:**

There was substantial heterogeneity between estimates of oral HPV prevalence in MSM populations that was partly explained by HIV status and median age.

## Introduction

Cancers of the oropharynx and tonsil, associated with HPV, have been increasing in recent decades [1] yet little is known about the risk of these cancers among men who have sex with men (MSM).[2] MSM have a disproportionately high prevalence of anogenital HPV infection and related disease compared to heterosexual men.[3–5] In the UK, the Joint Committee on Vaccination and Immunisation (JCVI) has recommended HPV vaccination of MSM but acknowledges substantial uncertainties about the burden of HPV-related disease in this group.[6–8] The potential impact of vaccination of MSM, or all males, on oral HPV infection and related disease cannot be fully assessed without quantifying preventable oral HPV infection and disease.

The epidemiology of oral HPV infection has recently been reviewed.[9] A systematic review of studies of oral HPV infection in healthy men and women was performed in 2010 which estimated the prevalence of oral HPV as 4.6% and 4.4% for men and women, respectively.[10] The prevalence in MSM was not considered separately in these reviews but given their higher prevalence of anogenital HPV, might be expected to have higher.

The natural history of oral HPV infection is not well understood and while HPV vaccine efficacy against oral HPV infection has been demonstrated [11] the impact of HPV vaccines on oral cancer and its precursors has not been studied. There has been no meta-analysis of the incidence and clearance rates of oral HPV infection, nor of risk factors for oral HPV acquisition. About 25% of head and neck cancers and 45–90% of oropharyngeal cancers are associated with HPV infection [12,13] yet HPV infection in non-malignant tonsil tissue is rare (<0.1%).[14] The portion of HPV-associated oropharyngeal cancers associated with HPV16 (87% [13]) is significantly more than that for anogenital cancers (66% for anal cancer [15]). The rate of disease progression in those with oral HPV infection and the nature and effect of co-factors remains uncertain.

We performed meta-analyses of (i) the prevalence of oral HPV infection in MSM; and (ii) the association between HPV infection in MSM compared to heterosexual men. Additionally, we reviewed incidence and clearance rates for oral HPV infection; risk factors for prevalence, incidence, and persistence; and concordance between oral and anogenital infection.

## Methods

### Search strategy and inclusion/exclusion criteria

A systematic review was performed of studies measuring HPV DNA prevalence, incidence and/or persistence in the oral cavity of MSM. We included studies in HIV-negative and HIV-positive populations, published in English, where an MSM-specific estimate was available in ≥ five MSM. We excluded case-control studies and clinical case series (e.g. transplant recipients, oral lesions) that might introduce additional heterogeneity to prevalence estimates.

For oral HPV prevalence estimates, we excluded conference abstracts that were superseded by journal articles and we included baseline estimates from longitudinal studies.

We searched databases on 20th October 2015 using the search terms mouth/oral /oropharangeal, HPV/ papillomavirus /papillomaviridae and man/ men /boy$/ adult /male$ /MSM/ "men who have sex with men"/ gay$ /homosexual$ /bisexual$. We searched Medline via PubMed and Medline, Embase and psycINFO using the Ovid platform (Version: OvidSP_UI03.13.01.101, SourceID 63482) using the search strategy in S1 Table. We also reviewed reference lists of included articles to validate our search strategy.

### Study selection

All screening and study selection methods were performed independently by EK and SO and discrepancies were resolved by consensus. Publications were screened by title, then, if not enough detail was available, by review of the abstract, and then the full text. Where data were not stratified by sex and sexual orientation, and both variables were reported in the publication (not a conference abstract), we contacted the corresponding author, via emails (initial and three week follow-up), for additional data. If there was no response we excluded the publication. Additional stratified data were obtained from four of the six authors contacted for this purpose.

### Data extraction

All data extraction was performed by EK (50% was validated by SO). Discrepancies were agreed by consensus. The following data were extracted where available: author name, year of publication, median age and range (years), study location, source of recruitment, HIV status, specimen collection method, and HPV DNA detection/genotyping assay(s). For studies included in the summary HPV prevalence estimation, the following additional data were extracted where available: the number of MSM in the sample and those with any HPV DNA detected, any high risk HPV types (HR-HPV), any low risk HPV types (LR-HPV) and, individually, the number with HPV-16, HPV-18, HPV-6, and HPV-11. For studies including both HIV-positive and -negative MSM, data were extracted separately for each group and treated as separate studies. For studies examining risk factors for oral HPV DNA statistically significant (p<0.05) risk factors in univariate analyses, factors included in multivariate models and factors that were statistically significant in multivariate models were extracted. Finally, for studies included in the meta-analysis of oral HPV prevalence in MSM compared to heterosexual men, the number of MSM and the number of heterosexual men with and without HPV were extracted.

We found little variation in the definitions used for HR-HPV and LR-HPV but substantial variation in HPV detection/genotyping assays (S2 Table). Definitions of MSM varied according to what was available in each publication and was based on either reported behaviours (any sex with men) or sexual orientation. We used median age to reduce the impact of skewness on the summary statistic for the age distribution. Where median age (for MSM strata, by HIV status) was not available, we contacted authors. If there was no response we used the mean age. We obtained median age estimates from 9 of the 11 authors contacted.

### Assessment of risk of bias

For studies included in the HPV prevalence estimation and analysis of risk factors for current HPV DNA detection we used the strengthening the reporting of observational studies in epidemiology (STROBE) checklist, for items to be included in reports of observational studies, which examines selection and information bias.[16] A count of checklist items was used as a marker for risk of bias in each study. Low risk of bias was assigned if ≥20 items were identified, medium if 15–19, and high if <15. STROBE count was explored as a potential source of heterogeneity using meta-regression.

### Statistical analysis

HPV DNA prevalence in MSM was determined as the percentage with detectable DNA in the total number of samples that were tested and adequate for PCR. For consistency, we calculated Clopper-Pearson 95% confidence intervals (CI) for each study superseding the published intervals. We did not expect the true population prevalence to be equal in all studies due to heterogeneity in study populations, specimen collection, and testing, so we used random effects meta-analysis to calculate a pooled estimate with 95% confidence interval. We quantified heterogeneity using the I^2^ statistic.

We performed a random effects meta-regression to examine the effect of HIV status, recruitment source (sexual health clinic [SHC], HIV clinic, community), oral specimen collection method (rinse/gargle alone or combination, other without rinse/gargle), STROBE count, and median age of participants on prevalence of HPV DNA and HR-HPV DNA. We included age and HIV *a priori* in a multivariate meta-regression model and other variables significant in univariate analyses. P values were obtained by comparing the multiparameter Wald test statistic to the appropriate F distribution with Knapp-Hartung modification to standard errors.

We calculated odds ratios (OR) to represent the effect of male same-sex activity on oral HPV prevalence within each study, and used random-effects meta-analysis to calculate a pooled OR and 95% CI. We examined the funnel plot of the inverse of the standard error and the effect to see whether the ORs across studies were associated with sample size.

We standardised estimates of incidence and clearance rates to the same unit (per 1000 person-months), and calculated these rates approximately if they were not presented but sufficient information was reported. For example if median duration of infection was given, in months, we assumed an exponential rate and calculated the rate of clearance as the inverse of median duration of infection multiplied by 1000. We calculated 95% CIs for these rates using the Poisson exact method. Given the heterogeneity introduced by different approximate methods of estimation, we did not perform a meta-analysis on these data.

All meta-analyses were conducted using either the metareg, metaprop [17] or metan functions downloadable from the Boston College Statistical Software Components (SSC) archive and used with Stata 13·1.

## Results

**Fig 1:**
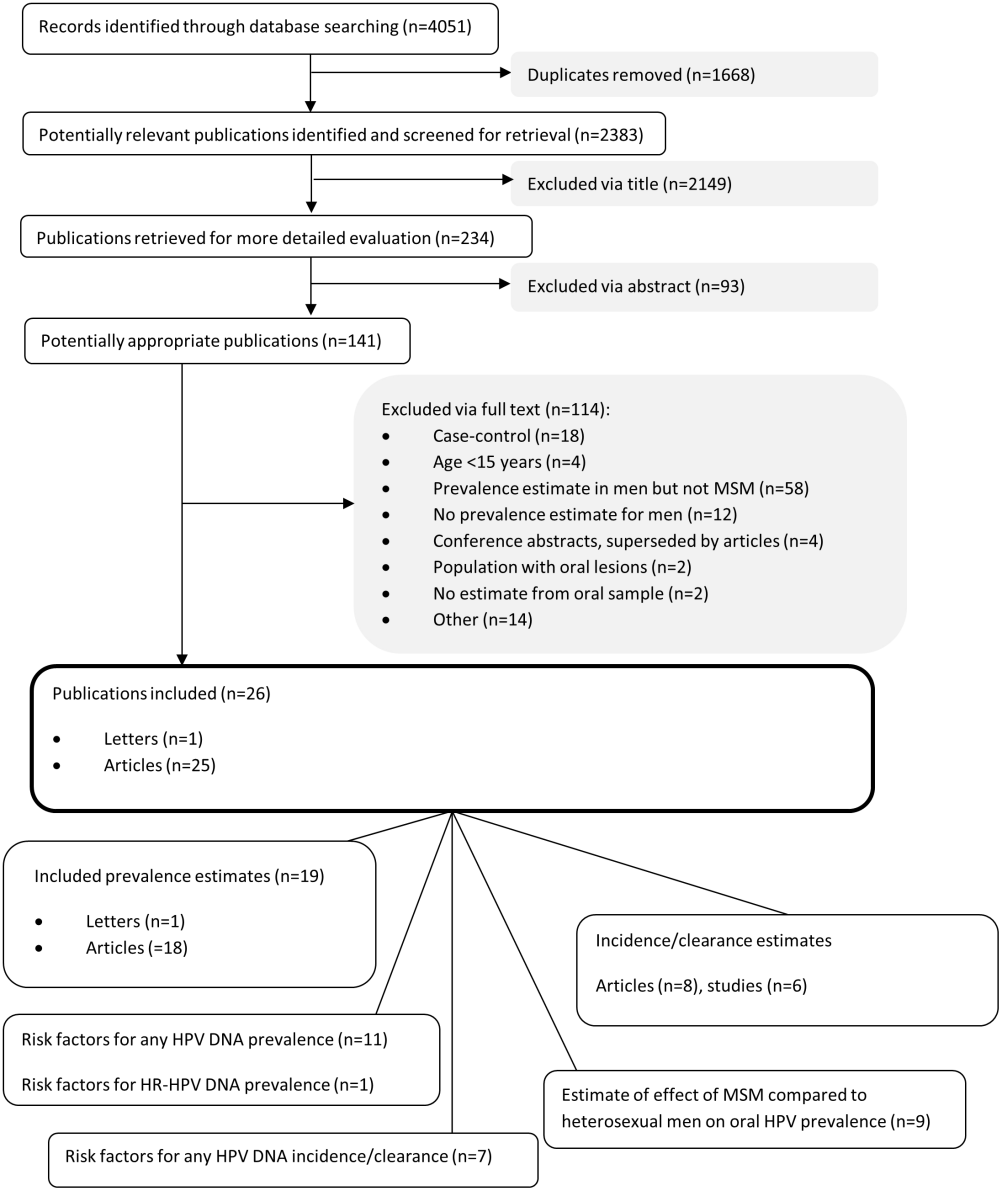
Flow diagram of screening and selection process.

2383 articles were identified and 2149 excluded based on information in the title. We excluded a further 93 based on information in the abstract and a further 114 having retrieved the full text (Fig 1). We included 26 articles and one letter representing 20 different studies. Details of the studies, including methods, risk of bias, number of MSM recruited and with HPV detected are shown in S2 Table.

### Oral HPV DNA prevalence

**Fig 2:**
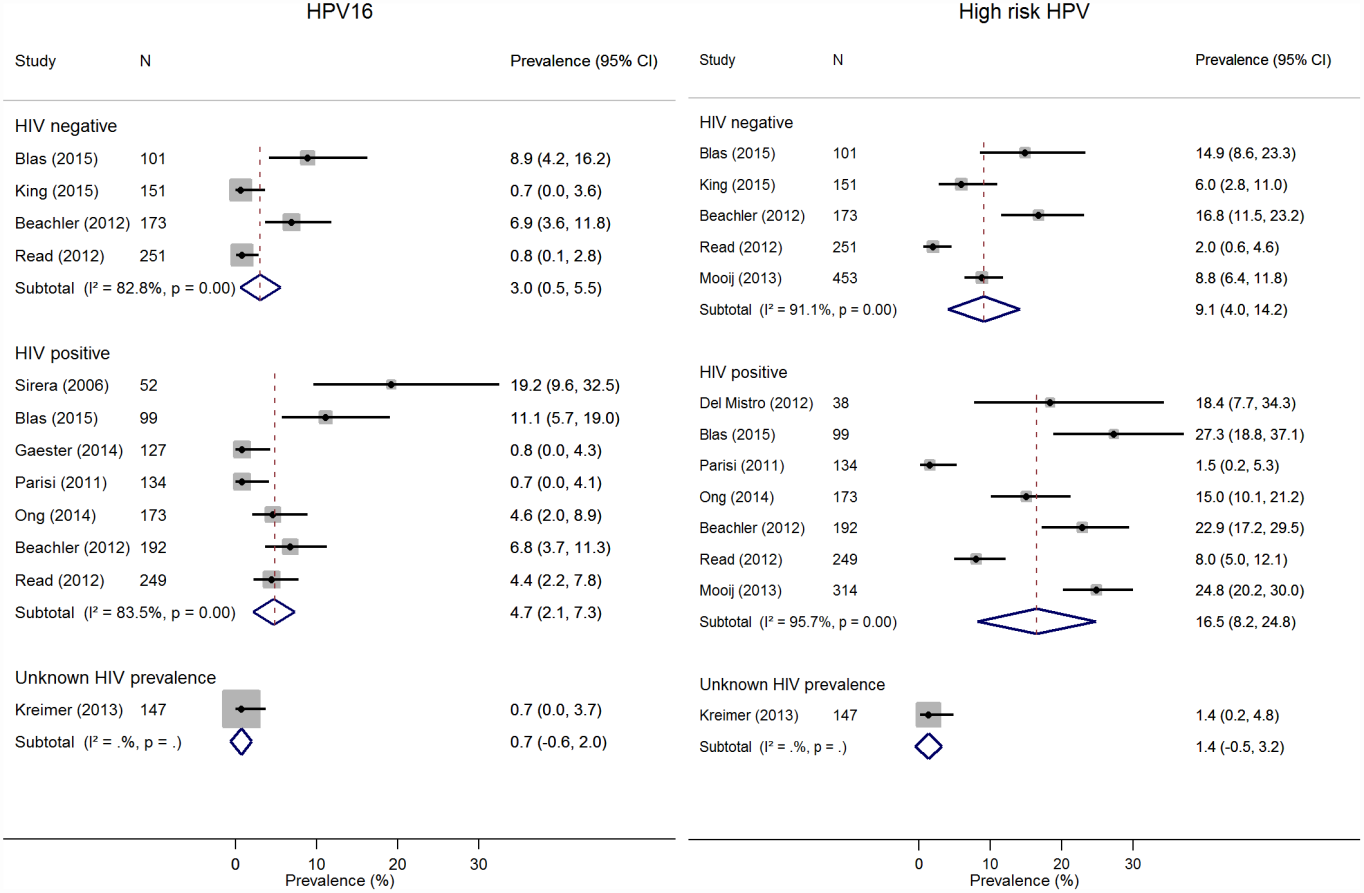
Random effects analyses of studies estimating oral HPV prevalence in MSM. Weights are from random effects analyses. Abbreviations: HPV=Human papillomavirus, MSM=men who have sex with men, HIV=Human immunodeficiency virus

For oral HPV DNA prevalence, we included 19 studies. Estimates of HPV DNA prevalence were available from six studies that included 1329 HIV-negative MSM,[18–22] 11 studies that included 1886 HIV-positive MSM,[18–20,22–29] and five studies that included 417 MSM with unknown HIV status[30–34]. For HPV16 prevalence, four estimates were available from HIV-negative populations[19,20,22,35] and seven from HIV-positive[19,20,22,28,36–38], in addition to one study with unknown HIV status[33]. For HR-HPV, there were five and seven estimates from HIV-negative[18–20,22,35] and HIV-positive[18–20,22,26,28,38] MSM populations respectively. Fig 2 shows that the random-effects pooled prevalence of HPV16 was 3.0% (95% CI 0.5–5.5) in HIV-negative and 4.7% (95% CI 2.1–7.3) in HIV-positive MSM. High heterogeneity was seen across studies in both HIV groups (I^2^ = 83% and 84%, respectively). The random-effects pooled prevalence of HR-HPV was 9.1% (95% CI 4.0–14.2%) in HIV-negative MSM and 16.5% (95% CI 8.2–24.8%) in HIV-positive MSM (I^2^ = 91% and 96%, respectively). S1 Fig shows that the pooled prevalence of any HPV was 17.1% (95% CI 7.3–26.8%) in HIV-negative and 28.9% (95% CI 19.1–38.7%) in HIV-positive MSM (I^2^ = 97% and 96%, respectively) and of any of the quadrivalent vaccine types HPV6/11/16/18 was 2.8% (95% CI 1.7–3.8%) in HIV-negative MSM and 5.9% (95% CI 0.8–11.0%).

**Fig 3:**
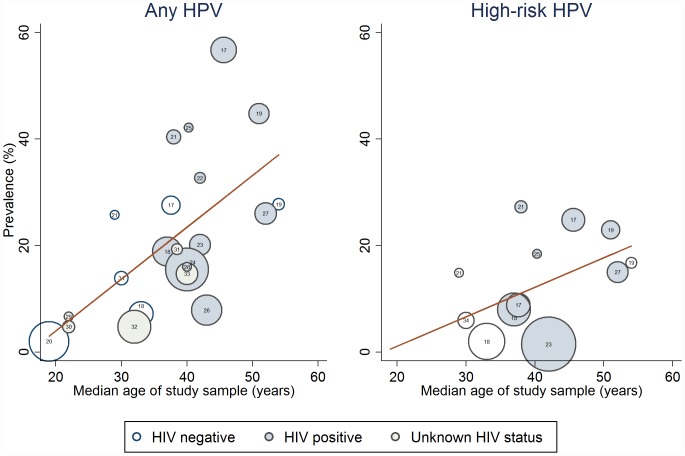
Meta-regression of median age of study population on study estimate for oral HPV DNA prevalence. Bubbles are weighted in size by inverse of within-study variance. Study reference represented by number within bubble.

As single covariates, source of recruitment (adjusted R^2^ = 3%, p = 0.30), mode of sample collection (adjusted R^2^ = -4%, p = 0.63), and STROBE count (adjusted R^2^ = -4%, p = 0.68) were not significant contributors to the heterogeneity of any HPV prevalence estimates (S3 Table). Heterogeneity in any HPV was in part explained by HIV status (adjusted R^2^ = 22%, p = 0.04) and by median age of study participants (adjusted R^2^ = 38%, p<0.01; Fig 3). Median age of study participants did not explain the heterogeneity in estimates of HR-HPV (adjusted R^2^ = 25%, p = 0.07) or HPV16 prevalence (adjusted R^2^ = 10%, p = 0.37). In multivariate analyses, median age remained a significant contributor to the heterogeneity of any HPV prevalence, after adjusting for HIV status (S3 Table).

The number of included studies was too low (<10 per stratum) to perform meta-regression in estimates from HIV-negative MSM or for HR-HPV and HPV16 estimates in HIV-positive MSM. In HIV-positive MSM there were 11 estimates of any HPV prevalence with high heterogeneity (I^2^ = 96%; S1 Fig) which was not explained by median age (adjusted R^2^ = 1%, p = 0.34), mode of specimen collection (adjusted R^2^ = -10%, p = 0.80) or STROBE count (adjusted R^2^ = 14%, p = 0.15). However recruitment source, especially recruiting from a community setting compared to an HIV clinic, was a significant contributor to heterogeneity (adjusted R^2^ = 65%, p = 0.01).

### Risk factors for oral HPV DNA detection

**Fig 4:**
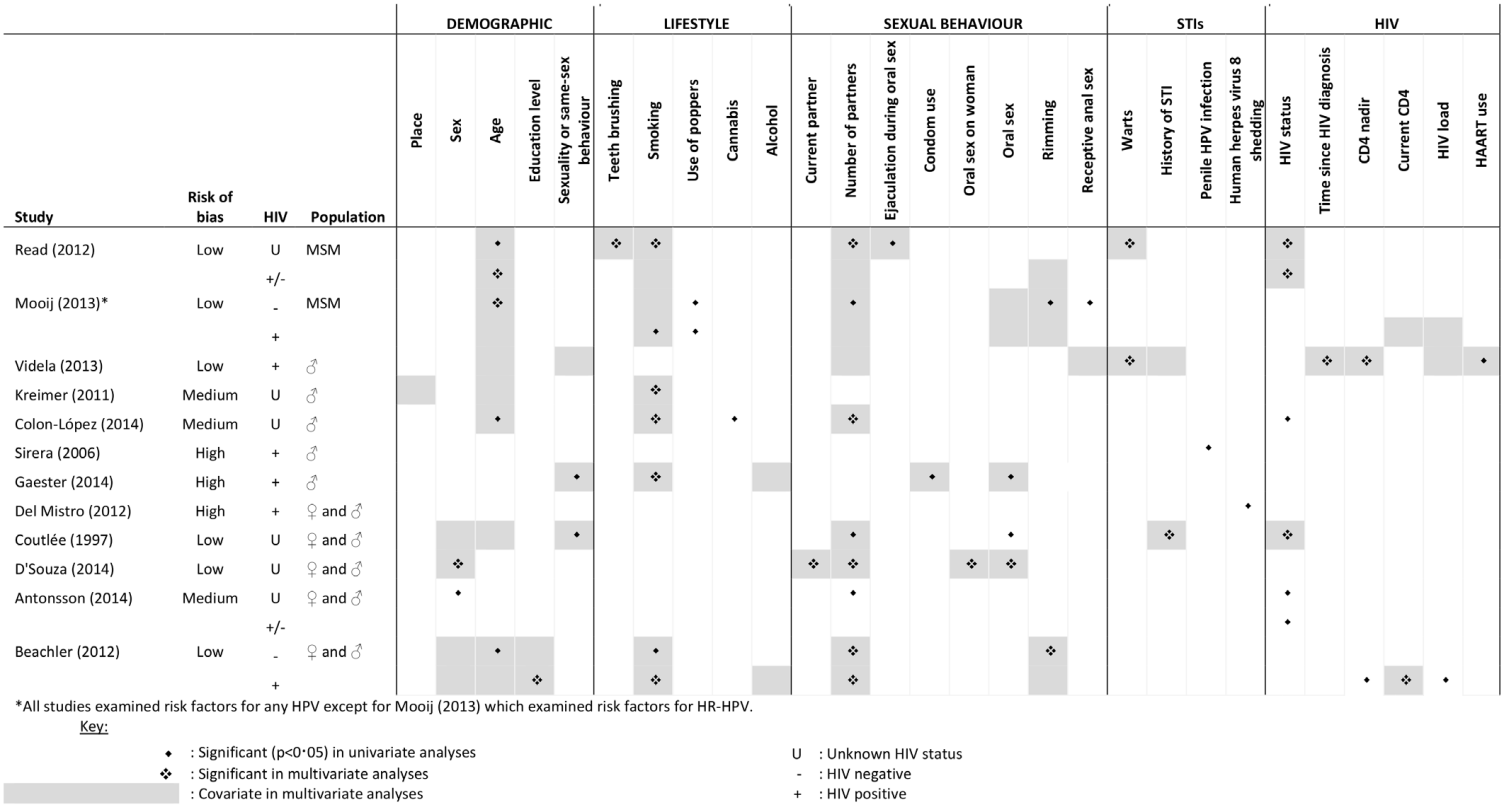
Studies examining risk factors for HPV DNA detection in the oral cavity that include MSM.

We analysed 12 studies that examined risk factors for oral HPV DNA detection and stated the number of MSM in the population (Fig 1). With the exception of Mooij *et al* who examined factors associated with HR-HPV,[18] all studies explored factors associated with any HPV. Fig 4 shows that the two studies within MSM populations [18,19] identified HIV infection, age (only HIV-negative MSM in Mooij *et al*), smoking (only HIV-positive MSM in Mooij *et al*), and number of sex partners (only HIV-negative MSM in Mooij *et al*) as risk factors. These were similar to the risk factors identified in studies that had, in addition to MSM, heterosexual male participants [23,25–27,32,39,40] and/or female participants.[20,26,39].

**Fig 5:**
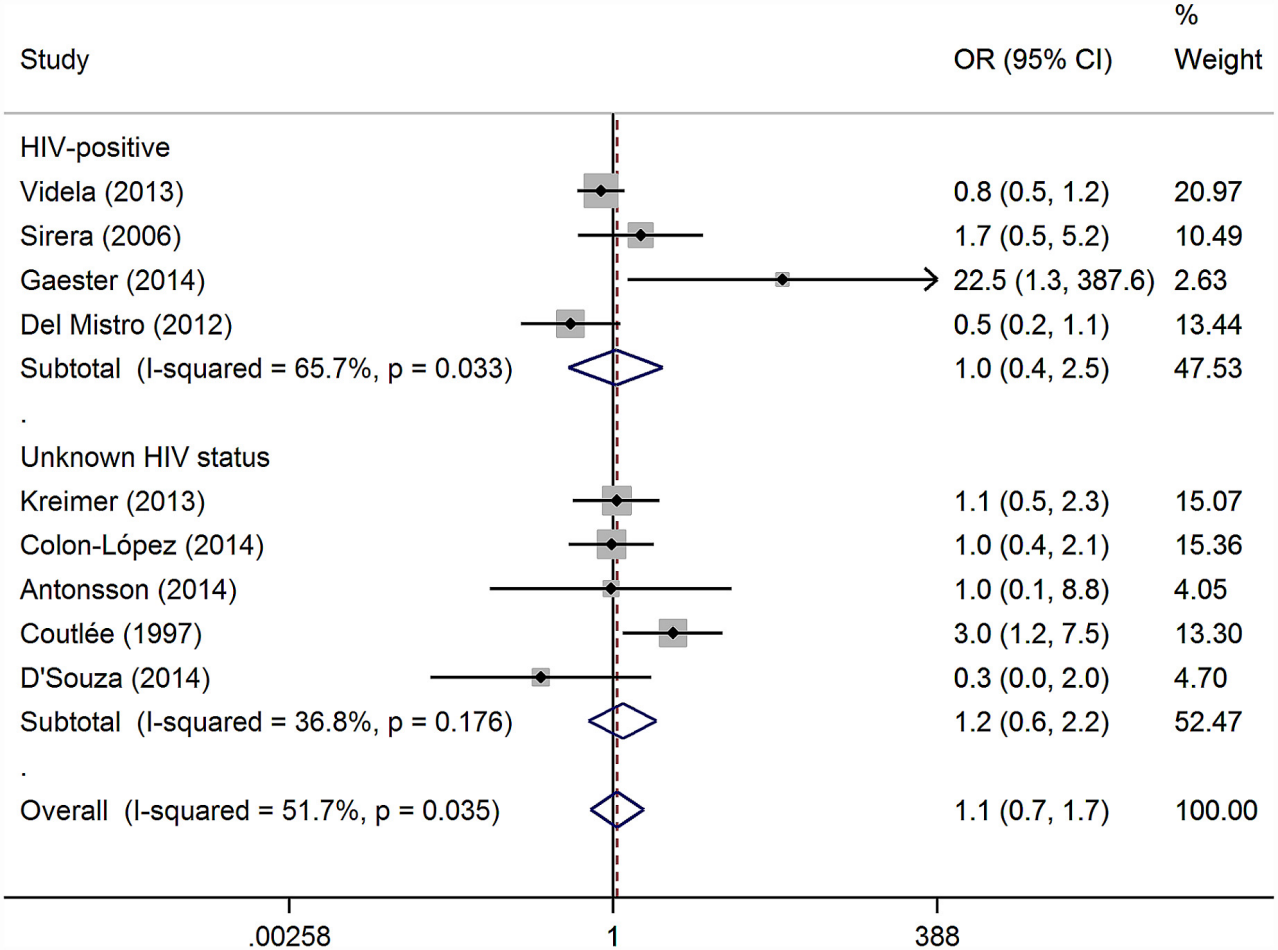
Studies examining the effect of having sex with men (and women) compared to having sex with women only on the prevalence of oral HPV. Weights are from random effects meta-analysis

Our meta-analysis showed no evidence that MSM were at higher risk of oral HPV DNA detection compared to heterosexual men (Fig 5). We included all nine studies measuring oral HPV in both MSM and heterosexual men. With heterosexual men as the reference group, meta-analysis resulted in a pooled OR of 1.07 (95% CI 0.65–1.74; p = 0.80), I^2^ = 51.7%, heterogeneity p = 0.04. There was little difference in effect size in the four studies in HIV-positive populations compared to the five with unknown HIV status and no studies in HIV-negative populations presented appropriate data to examine this effect. Excluding estimates from studies with a high risk of bias reduced the heterogeneity (I^2^) to 38.4% (heterogeneity p = 0.15) and gave a pooled OR of 1.05 (95% CI 0.65–1.68; p = 0.85). There were too few studies to formally test for small study bias.

### Oral HPV DNA incidence and clearance rates

We examined HPV DNA incidence and clearance rates in six longitudinal studies (Table 1). There was inconsistency in the reporting of rates, due to variability in study design, particularly visit schedule and follow-up period. Incidence of oral HPV in MSM ranged from 4 to 38 per 1000-person months. Men who reported sex with men and women had higher oral HPV incidence than men who exclusively had sex with men in the two studies where comparison was possible [33,39] but there was no difference in the probability of persistent infections.[39] In the study where direct comparison of HIV-positive and HIV-negative MSM was possible, incidence was higher in HIV-positive MSM (HPV16 IRR = 4.1; 95% CI 1.3–13.2; HPV18 IRR = 6.6; 95% CI = 1.4–30.8).[41,42]

**Table 1:** Incidence and clearance estimates of HPV DNA in oral cavity of MSM.

						(/1000 person-months)					
Study	Oral sampling interval(monthly)	Length of follow-up (months)	HIV status	Population	Sample size	INCIDENCE			CLEARANCE		
						Any HPV	HR-HPV	HPV16	Any HPV	HR-HPV	HPV16
H2M study: Van Aar (2014)[42]	6	12	-	MSM	433			0.9 (0.3–2.3)			NE
			+	MSM	290			3.5 (1.9–6.6)			NE
H2M study: Mooij (2014)[41]	3–6	6	-	MSM	413		6.9 (4.0–11.0)			115 (76–168)	54 (11–162)
			+	MSM	276		23.6 (16.8–32.3)			86 (59–120)	107 (49–203)
Beachler (2015)[43]	6	Median = 24.4 IQR = 18.4–35.4	-	MSM	220	12.3 (9.7–15.5)			NE		
			+	MSM	327	28.8 (25.3–32.6)			NE		
Videla (2013)[25]/ Darwich (2014)[54]	12	median = 24 IQR = 12–36	+	MSM	333	5.1 (3.5–7.0)		0·8 (0·3–1·7)	16.1 (10.7–23.1)		18.9 (9.4–33.8)
Ong (2014)[44]	36	36	+	MSM	249	4.0 (2.6–5.8)	2.7 (1.5–4.3)		12.4 (6.8–20.2)	15.2 (6.8–27.3)	14.5 (4.2–32.3)
Beachler (2013)[39]	6	median = 18.2 IQR = 6.2–24.0 max = 31.6	+	MSM	69	31 (NE-NE)			750 (733–767); 900 (866–935)*		
				hetero men	168	38 (NE-NE)					
Kreimer (2013)[33]	6	median = 12.7 IQR = 12.1–14.7 range = 0.3–37.2	U	MSM	54	4.1 (0.9–12.0)	2.7 (0.3–9.8)		175 (NE-NE)*+	175 (NE-NE)*+	
				MSMW	93	14.1 (7.9–23.3)	5.0 (1.9–11.0)		129 (NE-153)*+	153 (NE-192)*+	
				MSW	1392	5.1 (4.1–6.2)	2.4 (1.7–3.2)		154 (102–161)*+	159 (NE-167)*+	137 (NE-159)*+

HIV status: + = HIV-positive; − = HIV-negative; U = unknown.

*Clearance from incident infections only.

^+^Calculated from median duration of infection NE = Not possible to estimate.

IQR = Interquartile Range

Definitions of cleared HPV infection differed by study (single undetectable following a detectable or two consecutive undetectable following a detectable). In some studies only incident infections were ‘at risk’ for clearance, and clearance rate was reported in various ways, eg median duration of infection, percentage with persistent infection at six months, and number of incident infections clearing in 1000 person-months. However, clearance rate (range: 12-900/1000 person-months) was generally higher than acquisition rate.

Risk factors for oral HPV acquisition and persistence are described, but were not formally examined, due to the low number of longitudinal studies. In MSM overall in the Dutch H2M study and the US-based MACS cohort of MSM, HIV infection increased the risk of oral HPV acquisition[41–43]. Additional risk factors for incident oral HPV detection were identified in these two cohorts. In the MACS cohort, these included younger age, increasing education level, current alcohol use, number of recent oral sex partners and at least two recent rimming partners [43], and in the H2M cohort, increasing number of recent oral sex partners.[42]

For HIV-positive MSM in the MACS cohort risk factors were declining CD4+ T cell count, younger age and increasing number of lifetime oral sex partners, and in the H2M cohort, these were increasing number of recent oral sex partners, recent tobacco smoking and cannabis use.[42] On the other hand, for HIV-negative MSM in the MACS cohort risk factors were, increasing number of recent oral sex partners and having recently performed oral sex on a woman (aHR 2.8; 95% CI 1.1–7.2).[43]

In populations that included both MSM and non-MSM (and women [39,43]), HIV infection, including decreasing CD4+ T cell count, younger age, never having had a tonsillectomy[43] living in Mexico compared to USA,[33] smoking,[33] divorced/separated/widowed marital status,[33] increasing education level,[33] history of rimming,[39] bisexuality,[33] and female gender [39] were identified as risk factors for increased oral HPV incidence.

In MSM, oral HPV persistence was associated with increased time since HIV diagnosis [25,44] but not with HIV status.[42,43] Other factors identified that increase risk of HPV persistence were prevalent compared to incident HPV infections,[39,43] male sex, older age, lower CD4+ T cell count (men only) and current cigarette smoking (women only).[43]

### Concordance between oral and anogenital HPV infection

Four studies [19,24,35,45] presented data on HPV type-specific concordance at oral and anogenital sites, but only two in MSM where samples were taken at the same time: concordance was not found in 151 HIV-negative oral-anogenital pairs across 21 tested types [35] nor in 166 HIV-positive MSM across 25 tested types.[24] In heterosexual HIV-positive men, Videla *et al*. found that 2/191 (1%) had HPV-16 detected in the anal brushing, penile swab, and oral brushing.[25]

Concurrent HPV detection, without details of HPV type, at oral and anogenital sites in MSM was presented in two studies.[23,25] Videla *et al*. found that 65/458 (14%) MSM had concurrent oral and anal HPV infection and 30/457 (7%) had concurrent oral and penile.[25] Sirera *et al*. showed that penile HPV infection was related to oral HPV infection (OR 2·7; 95% CI 1·0–7·7) and that two or three site (penile, anal, oral) concordance of any HPV was 48%.[23] Blas *et al*, found 2/50 HIV-positive MSM had detectable HPV, but of different types, simultaneously in anal and coronal sulcus samples.[22] A further three studies were identified in which oral and anogenital samples had been tested for HPV but concordance estimates were not presented.[21,33,39]

## Discussion

The analysis of data from 26 publications has provided estimates of epidemiological parameters concerning oral HPV infection in MSM. These include prevalence, risk factors, and incidence and clearance rates. Substantial heterogeneity was identified between HPV prevalence estimates and subgroup analyses were performed by HIV status.

We showed a statistically significant contribution of HIV status to heterogeneity in oral HPV prevalence (of any type) across studies and the pooled estimate from this meta-analysis for HIV-negative MSM (any HPV = 16%; HPV16 = 2%) was half that for HIV-positive MSM (any HPV = 29%; HPV16 = 4%). Furthermore, HIV status was consistently found to be a risk factor for oral HPV infection in cross-sectional studies [18–20,32] and oral HPV incidence was three-fold higher in HIV-positive MSM than HIV-negative MSM in the only study where comparison was possible.[41] HIV and HPV epidemics interact because both infections are dependent on similar transmission behaviours and infection with one is thought to increase susceptibility to the other. HIV-induced immune defects also lead to an increased risk of reactivation from HPV latency, and delay in HPV clearance.[46,47]

We found a positive correlation between median age of study participants and prevalence which partly explained heterogeneity between study estimates. This association may be modified by HIV status since it was not found for prevalence estimates from HIV-positive populations. No within-study associations between age and oral HPV were found in studies of HIV-positive MSM, but four studies including HIV-negative MSM did find a statistically significant association with age [18–20,32] (only one in multivariate analysis)[18] and another found a non-significant trend.[40] This may suggest that oral HPV infection is independent of age in HIV-positive MSM but increases with age in HIV-negative MSM. However the HIV-positive MSM populations studied were generally older than HIV-negative populations so that an age-association in younger HIV-positive MSM may have been missed.

Pooled estimates of anal HR-HPV prevalence in HIV-negative (37.2%) and HIV-positive (73.5%)[48] MSM were four to five times higher than the pooled estimates from this meta-analysis for oral HPV prevalence. We assume that some reduction in oral prevalence is due to technical differences in sample collection, processing, storage, and testing especially as oral samples are particularly sensitive to sub-optimal DNA extraction methods.[49] However differences in epidemiological parameters such as incidence and persistence, and factors that underlie them, such as immune response, exposure, and transmission efficiency, should also be considered. Beachler *et al*. found that HPV incidence and persistence in MSM was higher at the anal site than oral and that heterosexual men were as likely to have persistent oral HPV but less likely to have persistent anal HPV.[39]

Oral HPV prevalence estimates in MSM from this meta-analysis were three to six times higher than the pooled estimate in all men (5%) from a previous systematic review [10] but there is no evidence from this analysis that MSM are more at risk of oral HPV infection than heterosexual men (although varying definition of MSM between studies and lack of data in HIV-negative MSM limits this conclusion). Women may play a role in oral HPV transmission in MSM who also have sex with women: in one study [33], compared to men exclusively having sex with women, oral HPV acquisition was higher in men who have sex with men and women, but not men having sex exclusively with men; in another study heterosexual men, but not MSM, were more at risk compared to women.[33,39]

Clearance rates were greater than incidence in all five longitudinal studies. The clearance rate applies only to MSM infected with HPV (10–30% of the MSM population) and incidence on the susceptible portion (70–90% of MSM, assuming no natural immunity) so that, all other things equal, in the total population the number of new infections will eventually equal the number of cleared infections and the prevalence will stabilise. For example, using rates from Videla *et al*, whatever the current prevalence, if there is no effective natural immunity and all other factors remain unchanged, the rates would equilibrate at a prevalence of 23% (empirical estimate was 15% in this population).[25]

The strengths of this study include the high inter-rater agreement in study selection and data extraction and the exploration of reasons for heterogeneity in prevalence estimates including risk of bias. Yet there remain several limitations. We were not able to fully explain the extreme heterogeneity between prevalence estimates so pooled estimates should be used with caution. We needed to use approximation methods to estimate incidence and clearance rates and standardise them across studies; this, undoubtedly, introduced uncertainty into the estimates and so precluded meta-analysis. Although not designed for measuring risk of bias in meta-analyses, we used the STROBE checklist, which was not ideal for assessing the quality of study designs. We were unable to include data from four potentially eligible studies.[50–53]

Considerable uncertainty remains in determining the public health implications of oral HPV DNA detection in MSM as it is not clear what proportion of infections will lead to cancer. However, these findings are important for our understanding of oral HPV epidemiology in terms of transmission, incidence, and persistence.

## Supporting Information

S1 Fig Random effects meta-analyses of oral HPV vaccine type DNA prevalence in men who have sex with men (MSM) (TIF)

S1 Table Search terms and strategy for Medline/Embase/PsychINFO via the Ovid platform and Pubmed. (DOCX)

S2 Table Characteristics of the studies and participants describing oral HPV prevalence, incidence, clearance rate, risk factors, and anogenital concordance in MSM. (DOCX)

S3 Table Meta-regression of oral HPV prevalence in MSM and study-related factors. (DOCX)

S4 Table PRISMA 2009 Checklist. (DOC)
