# Supplementary figures and images for: Oral Human Papillomavirus Infection in Men Who Have Sex with Men: A Systematic Review and Meta-Analysis
Source: PLoS One. 2016 Jul 6;11(7):e0157976. doi: 10.1371/journal.pone.0157976 (PMC4934925; doi:10.1371/journal.pone.0157976)

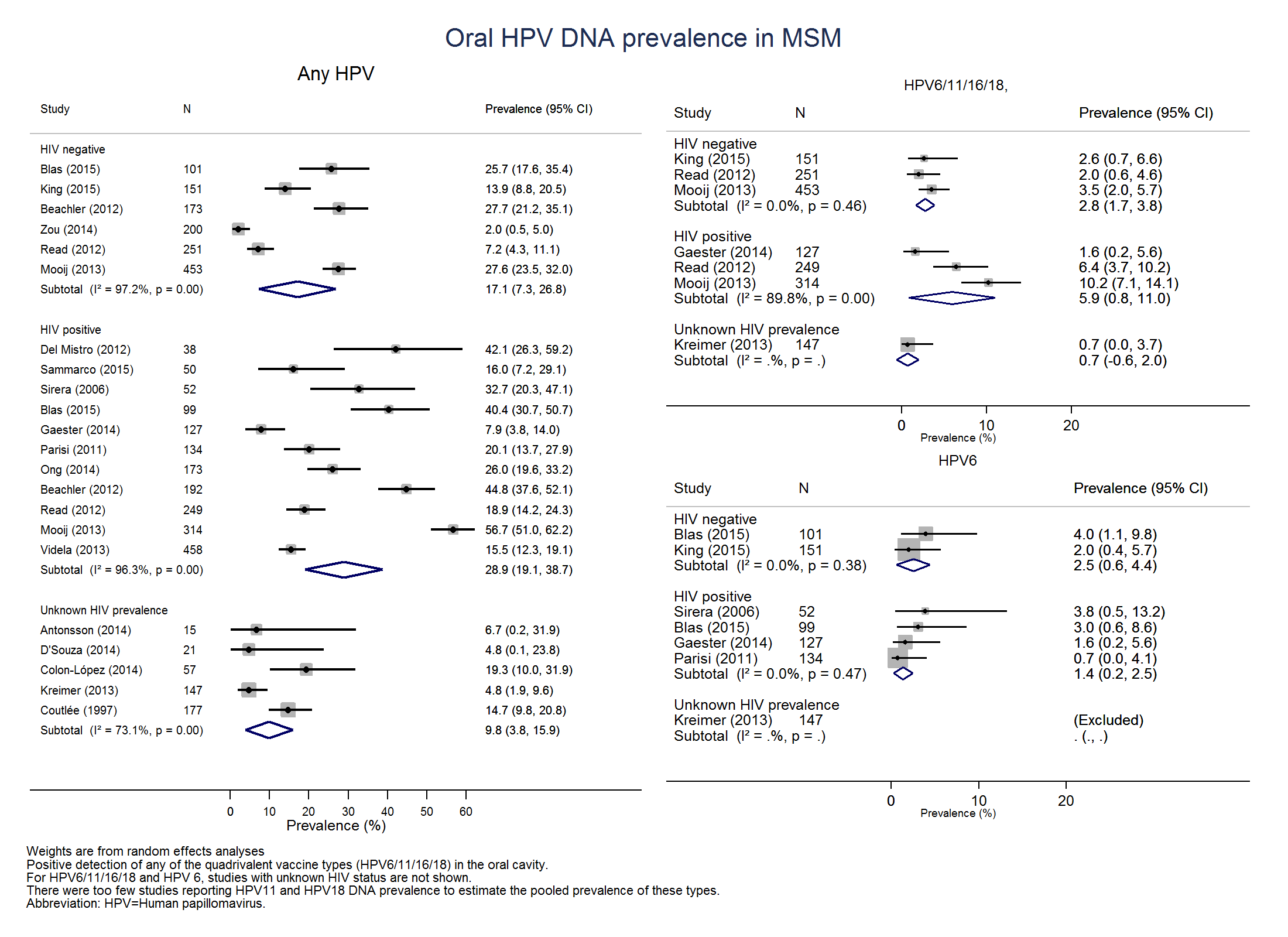

Supplement: S1 Fig — (TIF) [file pone.0157976.s001.tif]
