# Supplementary material for: Oral Human Papillomavirus Infection in Men Who Have Sex with Men: A Systematic Review and Meta-Analysis
Source: PLoS One. 2016 Jul 6;11(7):e0157976. doi: 10.1371/journal.pone.0157976 (PMC4934925; doi:10.1371/journal.pone.0157976)
Supplement: S1 Table — (DOCX) [file pone.0157976.s002.docx]

S1 Table. Search terms and strategy for Medline/Embase/PsychINFO via the Ovid platform and Pubmed

| Ovid platform search strategy | 1. ((mouth or oral or oropharangeal) and (HPV or papillomavirus or papillomaviridae) and (man or men or boy$ or adult or male$ or MSM or "men who have sex with men" or gay$ or homosexual$ or bisexual$)).mp. [mp=title, abstract, original title, name of substance word, subject heading word, keyword heading word, protocol supplementary concept word, rare disease supplementary concept word, unique identifier] |
| --- | --- |
|  | 2. limit 1 to english language |
|  | 3. remove duplicates from 2 |
| Pubmed user query | ((("mouth"[MeSH Terms] OR "mouth"[All Fields]) OR ("mouth"[MeSH Terms] OR "mouth"[All Fields] OR "oral"[All Fields]) OR oropharangeal[All Fields]) AND (HPV[All Fields] OR ("papillomaviridae"[MeSH Terms] OR "papillomaviridae"[All Fields] OR "papillomavirus"[All Fields]) OR ("papillomaviridae"[MeSH Terms] OR "papillomaviridae"[All Fields]))) AND (("men"[MeSH Terms] OR "men"[All Fields] OR "man"[All Fields]) OR ("men"[MeSH Terms] OR "men"[All Fields]) OR boy$[All Fields] OR ("adult"[MeSH Terms] OR "adult"[All Fields]) OR ("male"[MeSH Terms] OR "male"[All Fields]) or MSM or “men who have sex with men” or “gay$” or “homosexual$” or “bisexual$”) AND English[lang] |
