# Supplementary material for: Oral Human Papillomavirus Infection in Men Who Have Sex with Men: A Systematic Review and Meta-Analysis
Source: PLoS One. 2016 Jul 6;11(7):e0157976. doi: 10.1371/journal.pone.0157976 (PMC4934925; doi:10.1371/journal.pone.0157976)
Supplement: S2 Table — (DOCX) [file pone.0157976.s003.docx]

S2 Table. Characteristics of the studies and participants describing oral HPV prevalence, incidence, clearance rate, risk factors, and anogenital concordance in MSM.

|  |  |  |  |  |  |  |  |  |  |  |  | **Number of MSM** | | |
| --- | --- | --- | --- | --- | --- | --- | --- | --- | --- | --- | --- | --- | --- | --- |
| **Study/**  **publication** | **Analysis^a^** | **risk of bias ^b^** | **Study location** | **Oral specimen** | **DNA extraction** | **PCR amplification** | **Genotyping** | **HR-HPV definition** | **median Age (years)** | **Recruitment** | **HIV^c^** | **MSM** | **Any HPV detected** | **HR-HPV detected** |
| HIM |  |  | Mexico, Brazil, USA | rinse/gargle | Robotic MDx Media kit (Qiagen) |  | HPV Linear Array Genotyping test (Roche) |  |  | community/clinic |  |  |  |  |
| Kreimer (2011) | r |  |  |  |  |  |  | n/a |  |  | U | 130 | 4 |  |
| Kreimer (2013) | p, i/c | low |  |  |  |  |  | n/a | 32 |  | U | 147 | 7 | 2 |
| H2M |  |  | Amsterdam | rinse/gargle | MagNA Pure LC Total Nucleic Acid Isolation Kit (Roche) | DNA Enzyme Immuno Assay (HPV DEIA, Labo Bio-medical Products) | HPV LiPA25 (Labo Bio-medical products) |  |  | community/clinic |  |  |  |  |
| Mooij (2013) | p,r | low |  |  |  |  |  | A | 37.6 |  | - | 453 | 125 | 40 |
|  |  |  |  |  |  |  |  |  | 45.6 |  | + | 314 | 178 | 78 |
| Mooij (2014) | i/c (6 month) | |  |  |  |  |  | A | 38 |  | - | 413 |  |  |
|  |  |  |  |  |  |  |  |  | 47 |  | + | 276 |  |  |
| Van Aar (2014) | i/c (12 month) | |  |  |  |  |  | A | 38 |  | - | 433 |  |  |
|  |  | |  |  |  |  |  |  | 47 |  | + | 290 |  |  |
| van Rijn (2014) | relationship to seropositivity | |  |  |  |  |  | D | 38 |  | - | 441 |  |  |
|  |  |  |  |  |  |  |  |  | 46 |  | + | 306 |  |  |
| MAC |  |  | Baltimore, Chicago, Pittsburgh | rinse/gargle | magnetic bead automated platform (QIAsymphonySP, Qiagen) |  | PGMY09/11 & reverse blot hybridisation | C |  | community |  |  |  |  |
| Beachler (2012) | p,r | low |  |  |  |  |  |  |  |  | - | 173 | 48 | 29 |
|  |  |  |  |  |  |  |  |  |  |  | + | 192 | 86 | 44 |
| Beachler (2015) | i/c |  |  |  |  |  |  |  |  |  | - | 220 |  |  |
|  |  |  |  |  |  |  |  |  |  |  | + | 327 |  |  |
| CARH-MEN Can Ruti HIV+ Men cohort |  |  | Spain | combined cytobrush &gargle | Qiamp Viral DNA kit (Qiagen) |  | IVD-CE *F*-HPV typing (Molgentix) | B |  | HIV clinic |  |  |  |  |
| Videla (2013) | p, i/c | low |  |  |  |  |  |  | 40 |  | + | 458 | 71 |  |
| Darwich (2014) | i/c | | |  |  |  |  |  |  |  |  |  |  |  |
| Melbourne SHC cohort study |  |  |  | tampon-absorbed oral swab & rinse/gargle &swab | MagNA Pure LC Total Nucleic Acid Isolation Kit (Roche) | PGMY09/11 &PCR-ELISA detection protocol | HPV Linear Array Genotyping test (Roche) | B |  | STI clinic |  |  |  |  |
| Read (2012) | p,r | low | Australia |  |  |  |  |  | 33 |  | - | 251 | 18 | 5 |
|  |  |  |  |  |  |  |  |  | 37 |  | + | 249 | 47 | 20 |
| Ong (2014) (follow-up to Read) | i/c |  | Australia | rinse/gargle |  |  |  |  | mean=52 |  | + | 173 |  |  |
|  |  |  |  |  |  |  |  |  |  |  |  |  |  |  |
| HYPER study |  |  |  |  |  |  |  |  |  | community |  |  |  |  |
| Zou (2014) | p | low | Australia | rinse/gargle | magnetic bead capture: either VERSANT kPCR Molecular System SP (Siemens) or MagNA Pure 96 (Roche) | PGMY09/11 &PCR-ELISA detection protocol | HPV Linear Array Genotyping test (Roche) | n/a | 19 | community | - | 200 | 4 |  |
| Other |  |  |  |  |  |  |  |  |  |  |  |  |  |  |
| Beachler (2013) | i/c |  | Baltimore, US | rinse/gargle | Puregene DNA purification kit (Gentra systems) | PGMY09/11 & reverse blot hybridisation |  | C | 46 |  | + | 69 |  |  |
| Parisi (2011) | p | medium | Italy | oral swab | QIAamp DNA mini kit (Qiagen) & ExoSAP-IT (USB corp.) | MY09/MY11 primers followed by GP5+/GP6+ if negative | sequencing and analysis in NCBI BLAST | C | 42 | HIV clinic | + | 134 | 27 | 2 |
| Sirera (2006) | p,r | high | Spain | cytobrush | check canadas concordance paper |  |  | B | mean=42 | HIV clinic | + | 52 | 17 |  |
| Del Mistro (2012) | p,r | high | Italy | saliva | proteinase K/phenol/chloroform | MY09/MY11 primers followed by nested biotinylated GP5+/GP6+ if negative | direct sequencing or reverse line Blot using Consensus High Risk HPV genotyping kit (Qiagen) | A | 40.3 | HIV clinic | + | 38 | 16 | 7 |
| Colon-López (2014) | p,r | low | Puerto Rico | rinse/gargle | DNA purification from buccal cell protocol from Gentra PureGene kit (Qiagen) | INNO-LiPA HPV Genotyping Extra Amp | INNO-LiPA HPV genotyping Extra assay (Innogenetics) | n/a | mean=38.5 | STI clinic | U | 57 | 11 |  |
| Gaester (2014) | p,r | high | Sao Paulo | rinse | Illustra Tissue and Cells GenomicPrep Mini Spin Kit (Easton Turnpike) | MY09/MY11 primers &g gel electrophoresis | Papillomastrip, based on reverse blot technique(Operon Immune & molecular diagnostics) | n/a | 43 | HIV clinic | + | 127 | 10 |  |
| Ong (2014) | p | high | Australia | combined rinse/gargle, rinse/gargle after brushing, toothbrush | MagNA Pure 96 isolation and purification systmen (Roche) | PGMY09/11 &PCR-ELISA detection protocol | HPV Linear Array Genotyping test (Roche) or SPF10-LiPA 25 assay version | B | 52 | STI clinic | + | 173 | 45 | 26 |
| Coutlée (1997) | p,r | low | Canada | cytobrush | Lysed with Tween 20 and NP-40 | MY09/MY11 primers followed by spotting onto nylon membranes and HPV generic probelabelled with ^32^P-deoxynucleotides | type-specific oligonucleotide probes end-labelled with ^32^P-ATP | n/a | 40 | STI clinic/gastroenterology clinic | U | 177 | 26 |  |
| D'Souza (2014) | p,r | low | Baltimore, US | rinse/gargle | manetic bead-based automated QIASymphony SP (Qiagen) | PGM09/11 followed by reverse line blot hybridisation | HPV Linear Array Genotyping test (Roche) | C | 22 | STI clinic | U | 21 | 1 | 0 |
| Antonsson (2014) | p,r | medium | Brisbane, Australia | rinse/gargle | QIAamp DNA mini kit (Qiagen) QIAGEN supplementary protocol for isolation of DNA | GP5+/GP6+ agarose gel; Agencourt AMPure PCR purification kit | Sequencing followed by BLAST database | n/a | 22 | university | U | 15 | 1 |  |
| Blas (2015) | p | low | Lima, Peru | rinse/gargle | Qiamp DNA Blood mini kit | MY09/11 | HPV Linear Array Genotyping test (Roche) |  | 29 | Community | - | 101 | 26 | 15 |
|  |  |  |  |  |  |  |  |  | 38 |  | + | 99 | 40 | 27 |
| King (2015) | p | medium | London, UK | rinse/gargle | BioRobot Universal platform using QIAamp®DNA Blood BioRobot® MDx kit | in-house single-round multiplex PCR | based on the Bio-Plex® platform (Luminex xMAP®, Bio-Rad Laboratories) | B | 30 | STI clinic | - | 151 | 21 | 9 |
| Sammarco (2010) | p | high | Chieti, Italy | Swab | Maxwell16 viral total NA purification kit | Multiplex PCR, including MY09/11 | RFLP with results confirmed by sequencing |  | 40 | STI clinic | + | 50 | 8 |  |
|  |  |  |  |  |  |  |  |  |  |  |  |  |  |  |
| ^a^Analysis: publications included in prevalence study meta-analysis (p), risk factor analysis (r), incidence/clearance rates (ic) | | | | | | | | | | | | | | |
| ^b^HR-HPV definition: HPV16/18/31/33/35/39/45/51/52/56/58/59= A; with additional/68=B; with additional /68/73=C. HPV16/18/31/33/45/52/58=D  ^c^ HIV status: +=HIV-positive; -=HIV-negative; U=unknown | | | | | | | | | | | | | | |
