# Supplementary material for: Oral Human Papillomavirus Infection in Men Who Have Sex with Men: A Systematic Review and Meta-Analysis
Source: PLoS One. 2016 Jul 6;11(7):e0157976. doi: 10.1371/journal.pone.0157976 (PMC4934925; doi:10.1371/journal.pone.0157976)
Supplement: S3 Table — (DOCX) [file pone.0157976.s004.docx]

S3 Table. Meta-regression of oral HPV prevalence in MSM and study-related factors

|  |  | UNIVARIATE | | | | | MULTIVARIATE | | | | |
| --- | --- | --- | --- | --- | --- | --- | --- | --- | --- | --- | --- |
|  |  | **Coeff** | **Τ^2^** | **Residual I^2^ (%)** | **Adjusted R^2^ (%)** | **p value** | **Coeff** | **Τ^2^** | **Residual I^2^ (%)** | **Adjusted R^2^ (%)** | **p value** |
| HPV16 |  | | | | | | | | | | |
|  | **Multivariate model** |  |  |  |  |  |  | 17 | 83 | 46 | 0.80 |
|  | **HIV infection** |  | 15 | 83 | -29 | 0.64 |  |  |  |  |  |
|  | No | ref |  |  |  |  |  |  |  |  |  |
|  | Yes | 1.42 |  |  |  | 0.65 | 0.89 |  |  |  | 0.81 |
|  | unknown | -3.08 |  |  |  | 0.56 | -2.80 |  |  |  | 0.62 |
|  | **Median Age of study (years)** | 0.14 | 11 | 79 | 10 | 0.37 | 0.08 |  |  |  | 0.72 |
|  | **Recruitment source** | | 12 | 83 | 2 | 0.46 |  |  |  |  |  |
|  | HIV clinic | ref |  |  |  |  |  |  |  |  |  |
|  | STI clinic | -0.64 |  |  |  | 0.86 |  |  |  |  |  |
|  | community | 3.05 |  |  |  | 0.40 |  |  |  |  |  |
|  | **Oral specimen collection** | | 15 | 83 | -26 | 0.72 |  |  |  |  |  |
|  | Not rinse/gargle | ref |  |  |  |  |  |  |  |  |  |
|  | rinse/gargle | -1.47 |  |  |  |  |  |  |  |  |  |
|  | **STROBE count** | -0.05 | 15 | 83 | -23 | 0.88 |  |  |  |  |  |
| HR-HPV |  | | | | | | | | | | |
|  | **Multivariate model** |  |  |  |  |  |  | 62 | 94 | 17 | 0.23 |
|  | **HIV infection** |  | 64 | 95 | 13 | 0.19 |  |  |  |  |  |
|  | No | ref |  |  |  |  | ref |  |  |  |  |
|  | Yes | 6.85 |  |  |  | 0.20 | 4.26 |  |  |  | 0.45 |
|  | unknown | -8.04 |  |  |  | 0.39 | -6.36 |  |  |  | 0.49 |
|  | **Median Age of study (years)** | 0.55 | 55 | 93 | 25 | 0.07 | 0.64 |  |  |  | 0.07 |
|  | **Recruitment source** | | 67 | 95 | 10 | 0.26 |  |  |  |  |  |
|  | HIV clinic | ref |  |  |  |  |  |  |  |  |  |
|  | STI clinic | -0.49 |  |  |  | 0.95 |  |  |  |  |  |
|  | community | 8.09 |  |  |  | 0.29 |  |  |  |  |  |
|  | **Oral specimen collection** | | 77 | 95 | -4 | 0.54 |  |  |  |  |  |
|  | Not rinse/gargle | ref |  |  |  |  |  |  |  |  |  |
|  | rinse/gargle | 4.72 |  |  |  |  |  |  |  |  |  |
|  | **STROBE count** | **-**0.07 | 81 | 95 | -10 | 0.91 |  |  |  |  |  |
|  |  |  |  |  |  |  |  |  |  |  |  |
| Any HPV | **Multivariate model** |  |  |  |  |  |  | 128 | 94 | 36 | 0.01 |
|  | **HIV infection** |  | 157 | 96 | 22 | 0.04 |  |  |  |  |  |
|  | No | ref |  |  |  |  | ref |  |  |  |  |
|  | Yes | 11.65 |  |  |  | 0.09 | 4.78 |  |  |  | 0.49 |
|  | unknown | -7.10 |  |  |  | 0.38 | -5.33 |  |  |  | 0.47 |
|  | **Median Age of study (years)** | 0.97 | 125 | 93 | 38 | <0.01 | 0.74 |  |  |  | 0.04 |
|  | **Recruitment source** | | 195 | 97 | 3 | 0.30 |  |  |  |  |  |
|  | HIV clinic | ref |  |  |  |  |  |  |  |  |  |
|  | STI clinic | -7.39 |  |  |  | 0.39 |  |  |  |  |  |
|  | community | 3.80 |  |  |  | 0.65 |  |  |  |  |  |
|  | **Oral specimen collection** | | 209 | 97 | -4 | 0.63 |  |  |  |  |  |
|  | Not rinse/gargle | ref |  |  |  |  |  |  |  |  |  |
|  | rinse/gargle | -3.79 |  |  |  |  |  |  |  |  |  |
|  | **STROBE count** | 0.28 | 209 | 97 | -4 | 0.68 |  |  |  |  |  |
